# Bladder image stitching algorithm for navigation and referencing using a standard cystoscope

**DOI:** 10.1038/s41598-024-80284-7

**Published:** 2024-11-25

**Authors:** Ming Li, Nicole A. Varble, Sandeep Gurram, Dilara Long, Vladimir Valera, Nikhil Gopal, Ivane Bakhutashvili, Sheridan Reed, William F. Pritchard, John W. Karanian, Sheng Xu, Bradford J. Wood

**Affiliations:** 1 Center for Interventional Oncology, National Institutes of Health, Bethesda, MD USA; 2 Philips Healthcare, Cambridge, MA USA; 3 Urologic Oncology Branch, National Cancer Institute, National Institute of Health, Bethesda, MD USA; 4 Radiology & Imaging Sciences, Clinical Center, National Institutes of Health, Bethesda, MD USA; 5 National Institute of Biomedical Imaging and Bioengineering, Bethesda, MD USA

**Keywords:** Bladder map, Image stitching, Cystoscope, Cystoscopy, Endoscopy, Image processing, Bladder cancer

## Abstract

To aid in the diagnosis, monitoring, and surveillance of bladder carcinoma, this study aimed to develop and test an algorithm that creates a referenceable bladder map rendered from standard cystoscopy videos without the need for specialized equipment. A vision-based algorithm was developed to generate 2D bladder maps from individual video frames, by sequentially stitching image frames based on matching surface features, and subsequently localize and track frames during reevaluation. The algorithm was developed and calibrated in a 2D model and 3D anthropomorphic bladder phantom. The performance was evaluated in vivo in swine and with retrospective clinical cystoscopy video. Results showed that the algorithm was capable of capturing and stitching intravesical images with different sweeping patterns. Between 93% and 99% of frames had sufficient features for bladder map generation. Upon reevaluation, the cystoscope accurately localized a frame within 4.5 s. In swine, a virtual mucosal surface map was generated that matched the explant anatomy. A surface map could be generated based on archived patient cystoscopy images. This tool could aid recording and referencing pathologic findings and biopsy or treatment locations for subsequent procedures and may have utility in patients with metachronous bladder cancer and in low-resource settings.

**Supplementary Information:**

The online version contains supplementary material available at 10.1038/s41598-024-80284-7.

## Introduction

Cystoscopy plays a central role in the diagnosis, treatment, and follow-up for patients with bladder cancer and other pathologies. Evaluations, reports, and re-evaluation of specific bladder locations identified under cystoscopy require a high degree of spatial awareness and training. Cystoscopic views can be limited due to variable bladder volume and anatomy and may lead to incomplete surveillance, which further complicates sequential localization of specific regions of interest upon re-evaluation, especially for inexperienced operators.

During cystoscopy, specific regions of interest are often documented with generic two-dimensional (2D) bladder diagrams^1^, which lack spatial context or detailed and reproducible location descriptors. The inability to provide a comprehensive record and standardized reference images poses challenges in communication between operators, making re-identification of prior resection margins or areas of interest difficult. Full video files provide optimal spatial context but are not included in medical records and are not easily transmittable. While structured reporting software can improve the level of care^2^, a standardized method to fully convey 3D cystoscopy spatial information outside of verbal or written reports has not yet been adopted.

To overcome this, image stitching algorithms and endoscopic tracking techniques have been developed^3–10^. Such tracking methodologies may benefit less experienced users and improve inter-operator communication, enabling standardized and reproducible navigation guidance. In low-resource settings, the ability to reduce inter-operator variability and allow remote experts to view or compare results is particularly advantageous. Proposed methods for generation of 3D anatomic atlases^3–7^, mosaic or panoramic images, which are more easily shared^8,9^, would otherwise require specialized tracking systems, equipment, or high-resolution cameras^10^.

**Fig. 1:**
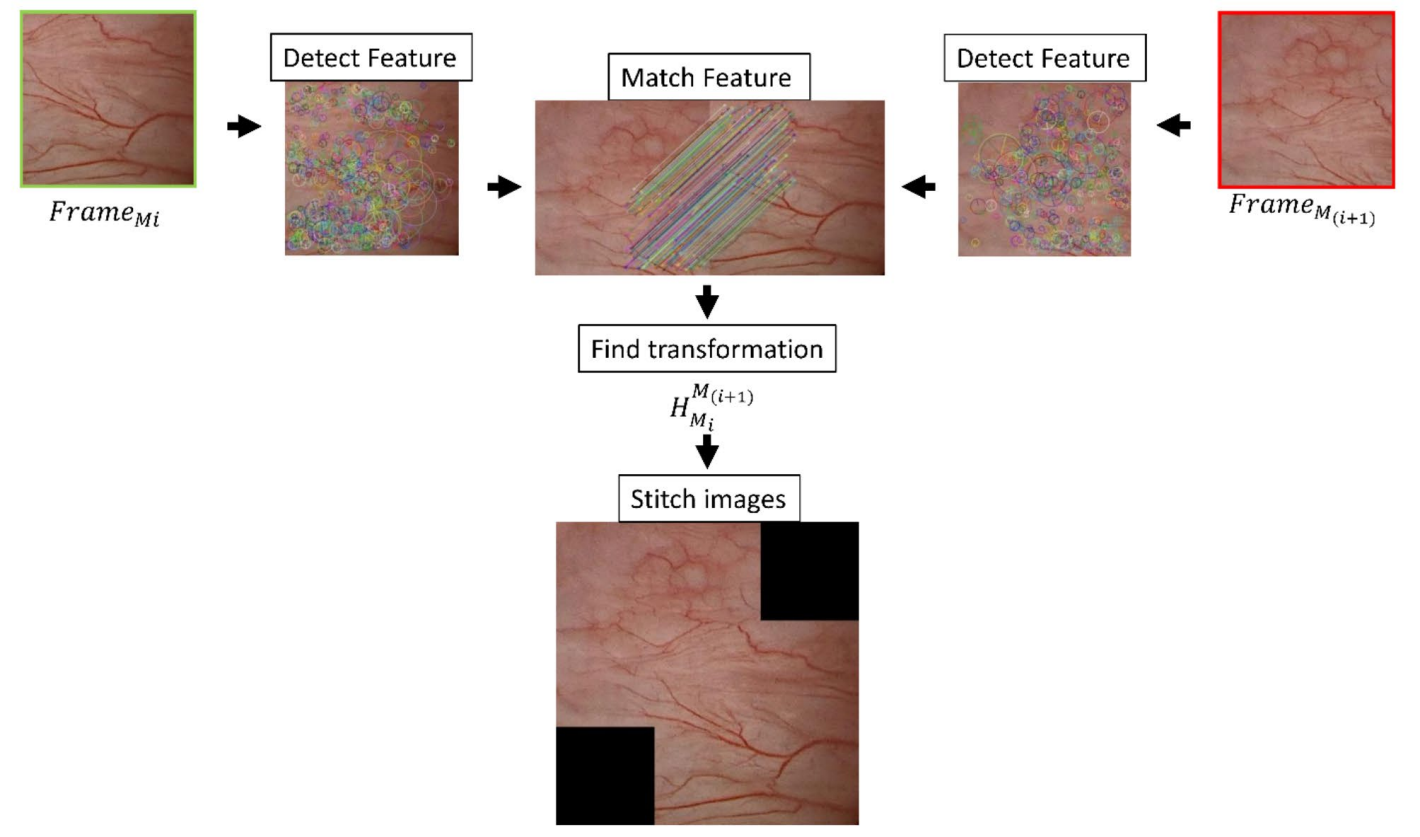
Bladder image stitching algorithm based on feature detection and matching of sequential images. Features are detected in the initial frame ($\:{Frame}_{{M}_{i}}$), and subsequent frame ($\:{Frame}_{{M}_{(i+1)}}$), matched, and the transform ($\:{H}_{{M}_{i}}^{{M}_{(i+1)}}$) is calculated to form a stitched, composite image

**Fig. 2:**
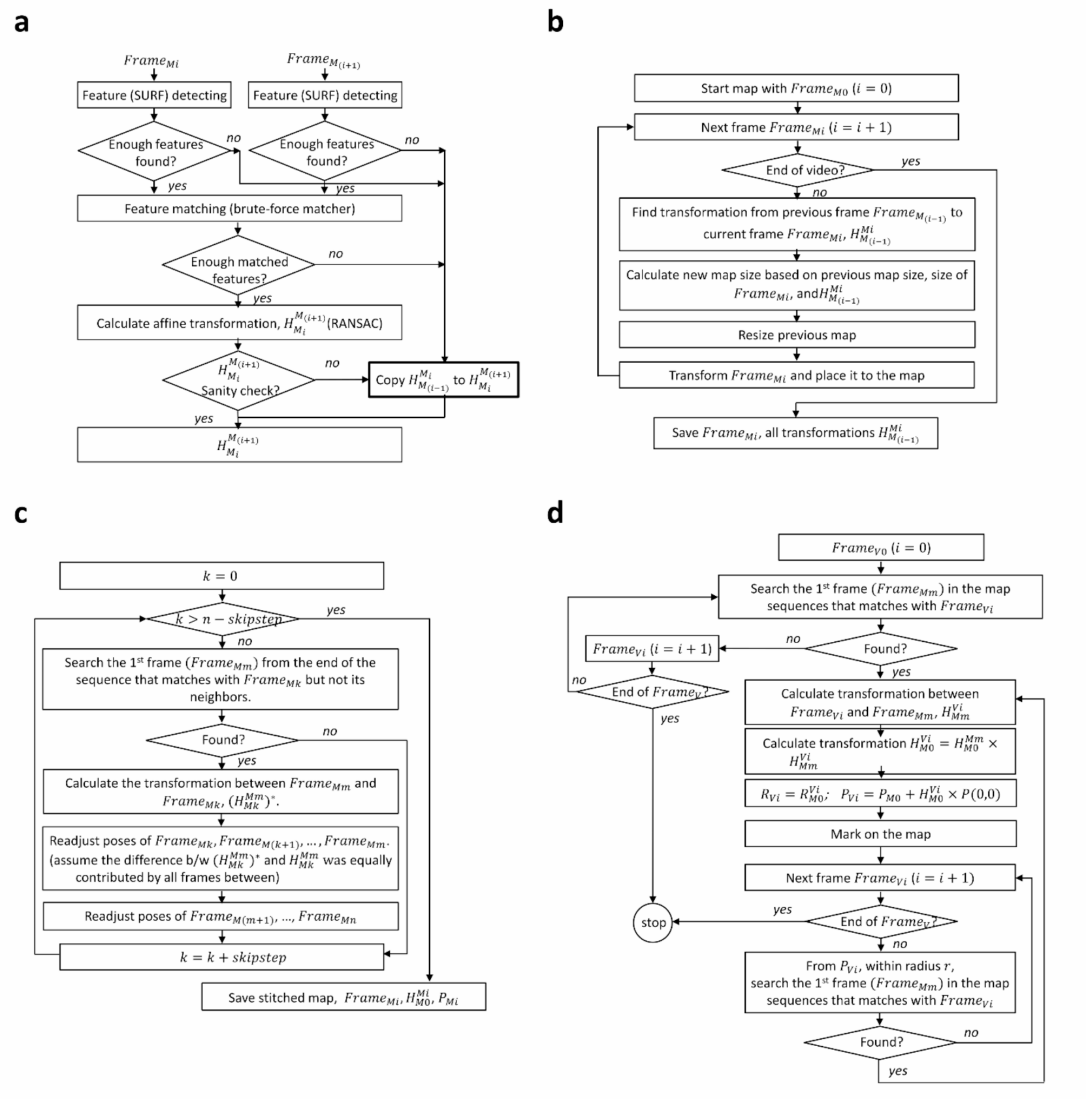
Flowchart description of the bladder stitching algorithm. Description of (**a**) feature detection and image transform calculation, (**b**) bladder map generation, (**c**) drift correction algorithm and (**d**) “revisit” algorithm, which enables video frames from repeat cystoscopy to be oriented to an original bladder map.

**Fig. 3:**
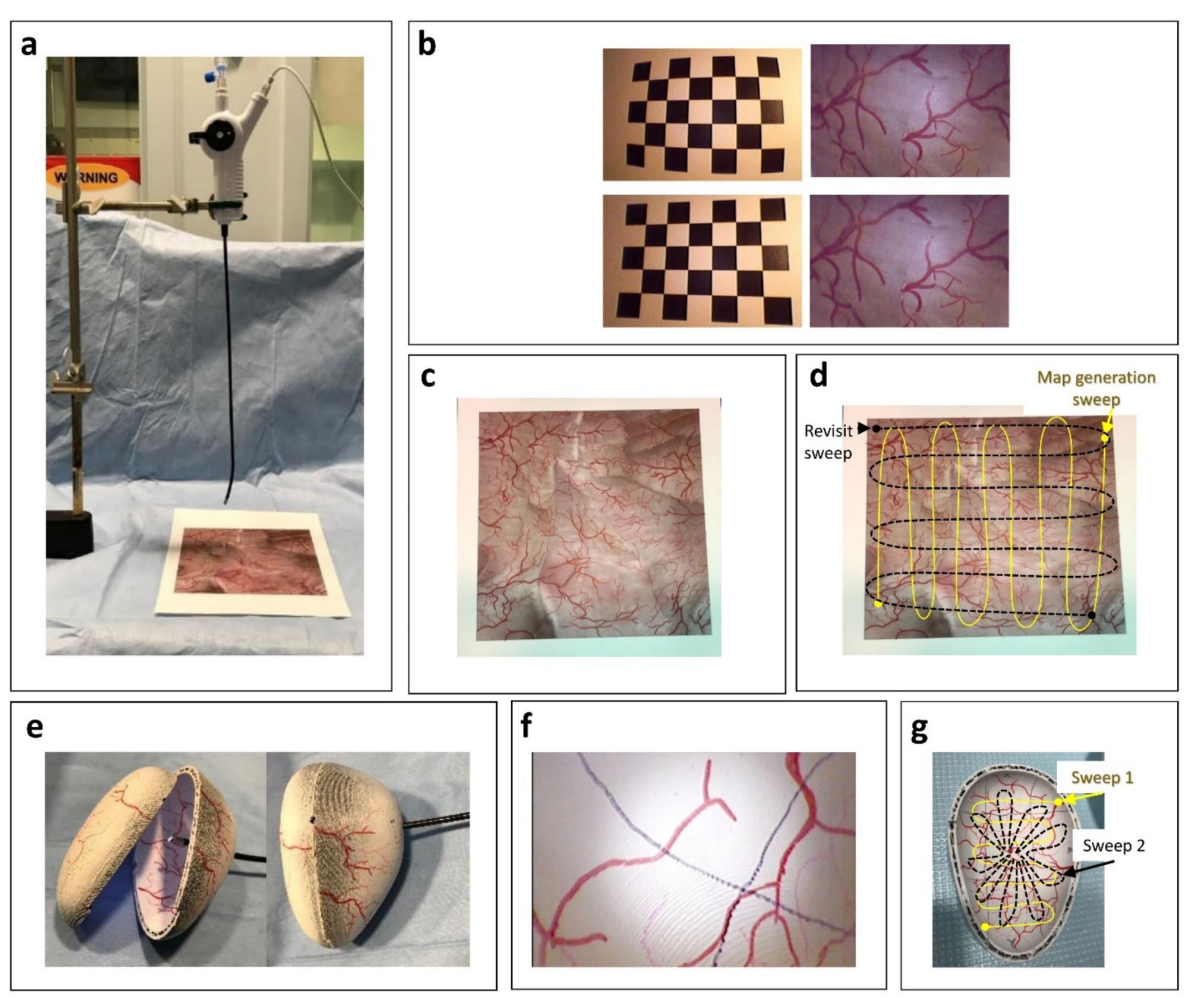
2D bladder image and 3D bladder phantom used in algorithm development and testing. (**a**) 2D experimental setup, (**b**) image calibration to correct for distortion and “fisheye” appearance with uncorrected (top) and corrected images (bottom), (**c**) test bladder image used in the 2D study, (**d**) illustrated sweep patterns used for original bladder map generation and revisit sweep, (**e**) 3D phantom, (**f**) example cystoscope image of 3D phantom, (**g**) illustration of two different sweep patterns performed to generate bladder maps, sweep 1 (yellow line) and sweep 2 (dashed black line).

Therefore, we developed an image stitching algorithm that aggregates and orients frames from videos to create a 2D representative map of a visualized field, while maintaining lesion orientation and frame of reference. Importantly (Figs. 1, 2 and 3), the image stitching process requires no hardware or specialized equipment and can use images recorded from the cystoscopy session without changing standard workflows. The algorithm was developed in 2D and 3D phantoms, tested in vivo in swine, and finally verified with human data. This technology has the potential to facilitate referencing and documentation in repeat cystoscopic procedures via enhanced tracking and localization, which are critical in longitudinal management of metachronous bladder lesions.

2D image accuracy was tested using an 18 cm × 18 cm bladder image placed approximately 10 cm away from the cystoscope lens. Two perpendicular sweep patterns were made to cover the entire image by moving the image as illustrated in Fig. 3a.

## Results

**Fig. 4:**
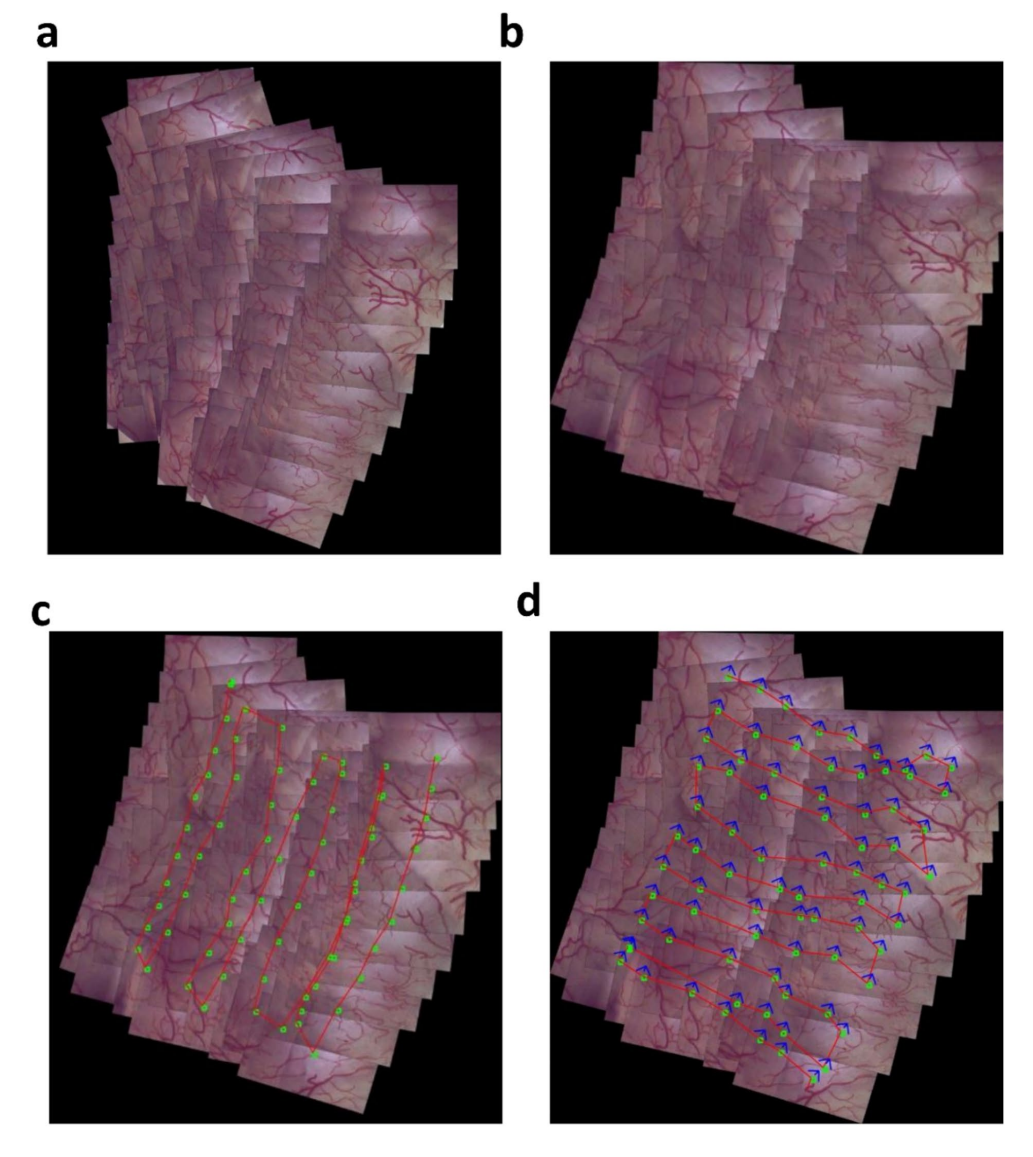
Bladder map generation from a 2D bladder image. Bladder map (**a**) before and (**b**) after drift correction. (**c**) Cystoscope sweep pattern used in bladder map generation. (**d**) Revisit sweep on the original, drift-corrected sweep. The red line indicates the pattern of the revisit sweep, the green dots indicate the predicted location of the cystoscope within the map, and the blue arrows indicate the predicted rotation of the cystoscope with respect to the initial frame of the bladder map (top-most right frame).

On both 2D and 3D phantom models, images were accurately stitched and used to aid localization of specific features during revisits, or follow-up cystoscopy, which implemented a distinct sweeping pattern. In the single study of the 2D phantom, a stitched map was created using 73 video frames (Fig. 4a). and drift correction was applied (Fig. 4b). Different from the original sweep pattern (Fig. 4c), the revisited sweep included 80 frames. It took 7 s to locate the position of the first image in the 2D map and an average of 0.76 s to localize successive frames. Image surface features were identified and mapped in 79/80 (99%) of frames. As shown through the localization and correct orientation of the cystoscope, the algorithm was able to accurately identify the location of an image in the 2D phantom, supporting the ability to reference to prior locations and navigate to lesions or tumors upon a repeat assessment (Fig. 4d).

**Fig. 5:**
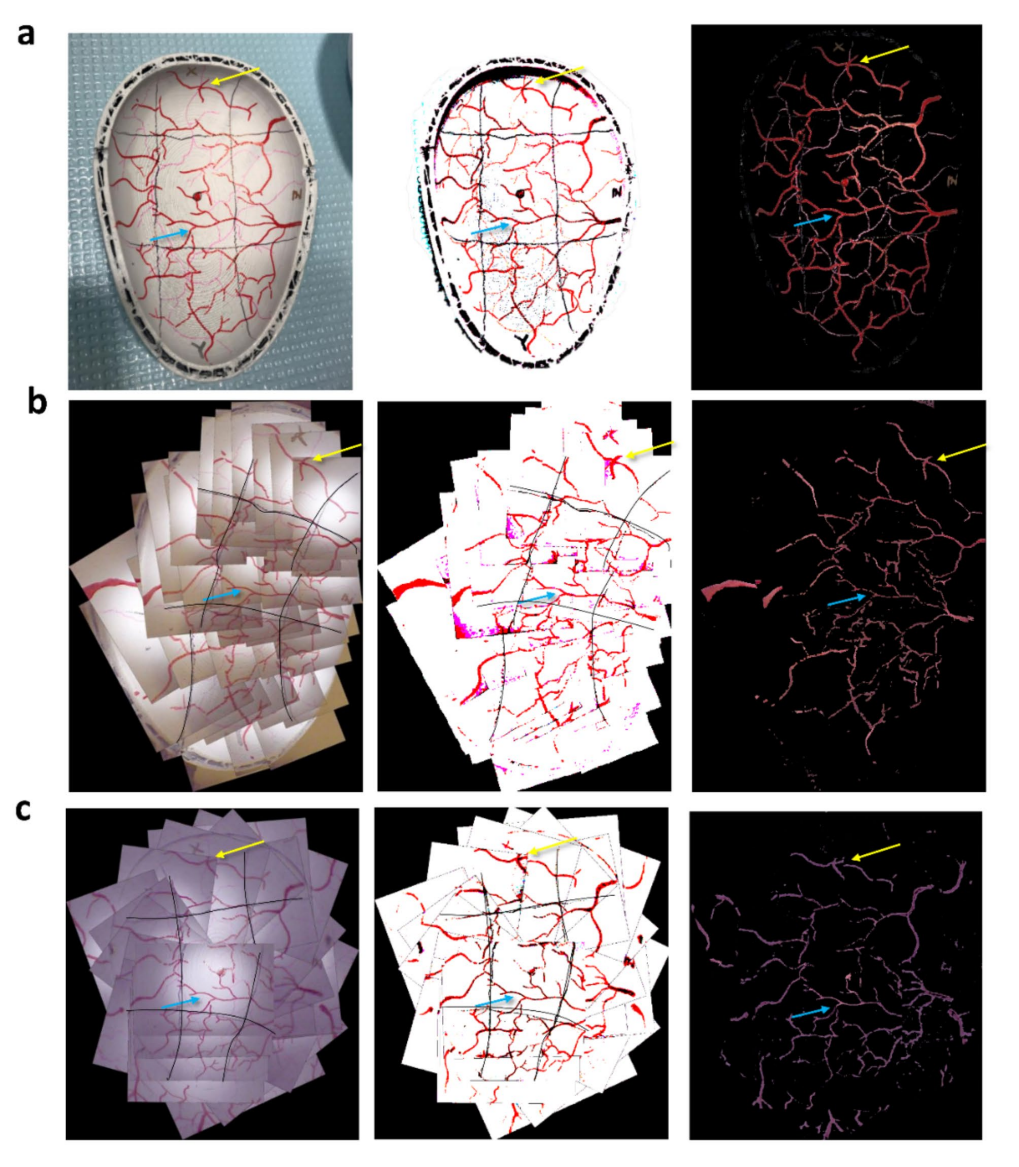
Assessment of bladder mapping algorithm on 3D bladder phantom. The left column is unprocessed, middle column is filtered to eliminates background shadows, and the right column is filtered to show just the vascular patterns. Blue and yellow arrows highlight common bifurcations between the original and stitched and processed images that highlight boundaries and vessels. (**a**) 3D bladder phantom with lines separating the half-bladder into 9 segments. (**b**) Images stitched together after a longitudinal sweeping image capture (sweep 1 illustrated in Fig. 1g). (**c**) Images stitched together after rotational image capture (sweep 2 illustrated in Fig. 1g).

Using a single sweep of the 3D phantom, 37 frames were acquired to create a map of half the bladder. A subsequent sweep, used for evaluating the ability to revisit locations in the initial map, generated a full map using 30 frames (Fig. 5). It took 4.5 s to localize the position of the first image frame and an average 0.35 s for each successive frame. 28/30 (93%) frames had sufficient image features to perform feature matching.

**Fig. 6:**
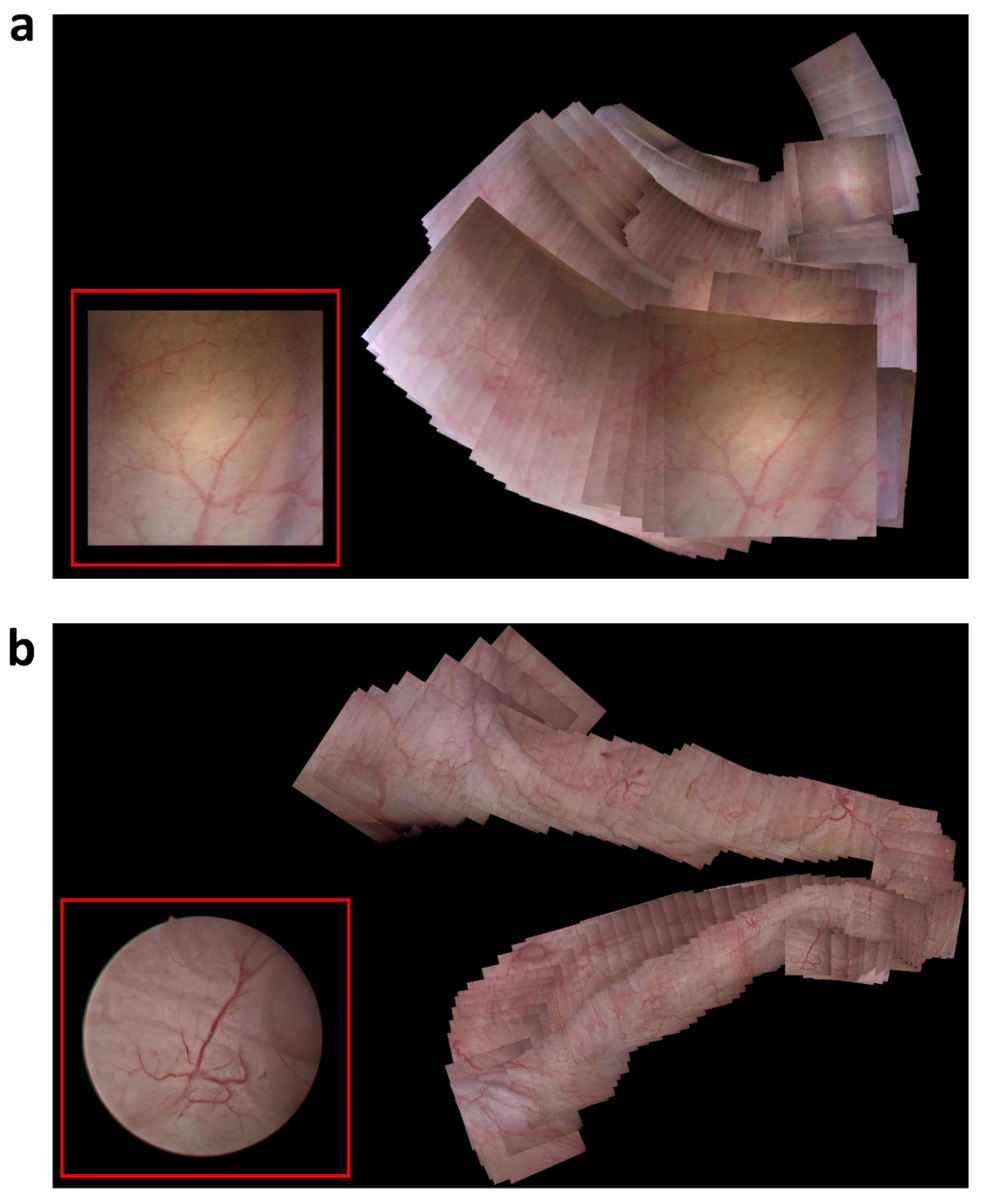
Stitched bladder maps from (**a**) in vivo porcine subject with disposable, flexible cystoscope, and (**b**) in vivo patient data. A single, unprocessed cystoscope frame is shown in bottom left of each image.

In preclinical data, 294 frames were acquired to generate a bladder map. Sufficient imaging features were identified and mapped in 271/294 (92%) frames. The bladder map was generated in 8 s. In clinical data, a high-quality cystoscope (image size 730 × 730 pixels) was used to generate the bladder map in 168 s (Fig. 6, Supplemental Video 1). The resulting video was 33 MB and the size of final frame was 9837 × 8585 pixels. 161/168 (96%) frames had sufficient imaging features to perform feature matching. While the relative number of frames with a sufficient number of surface features were similar across all studies and cystoscopes, we did find the number of seconds to localize the first image was longer in clinical data when using the high-quality rigid cystoscope, which is related to the higher resolution and number of image frames.

## Discussion

Bladder cancer is among the top ten leading causes of cancer-related deaths worldwide^11^ and presents a significant lifetime treatment cost, due to its metachronous nature, high rate of recurrence, and need for longitudinal surveillance^12,13^. Bladder cancer incidence is speculated to be higher in nations with high developmental index; however, high incidence of mortality is noted across North Africa in men and sub-Saharan Africa in women^14^. Low-middle income countries and rural areas of the United States may reflect varying and poor degrees access to expert care such as skilled cystoscopy^15^. Urothelial cancer care reflects a geographical health disparity given it requires complex access to multi-disciplinary specialists as well as technology and cystoscopy expertise. These geographical differences may be attributable to in part to differences in access to care and availability of diagnostic cystoscopy^16,17^.

We developed a tool that could help to standardize cystoscopy, enhance access and reproducibility, and reduce underdiagnosis with a focus on the ability to reference and revisit specific anatomic sites of concern during follow-up. Image stitching algorithms have been widely explored with endoscopic imaging sequences^10^, with urology and bladder being one of the most investigated due to the relatively predictable shape and feature-rich surface anatomy. Despite this effort, no image stitching algorithm has been deployed clinically. This may be due to the requirement for specialized tracking equipment or a limitation of applicable devices. Some solutions may only work with high-resolution cameras^5^, rigid cystoscopes^4,5^, tracking equipment^3,5,6^, or specialized imaging such as photodynamic diagnosis and tumor fluorescence^18^. The algorithm presented here was developed with disposable, flexible cystoscopes, whose use does not alter standard workflow, and is a low resource cost relative to these other technologies.

An additional goal of the algorithm presented in this study was to enable standardization, documentation, and sharing of results and data. Many image stitching algorithms aim to generate a 3D bladder model, which give a better understanding of the anatomical view of the bladder^3,4,6,19,20^, however, this limits the ability to share and quickly interpret cystoscopic findings. Kriegmair et al.^8^ and Groenhuis et al.^9^ demonstrated the ability to generate either 3D atlases or 2D maps of the bladder with Groenhuis et al. further demonstrating the ability to automatically detect feature changes in successive cystoscopic videos. As with those examples, the tool presented in this study permits sharing of single images with localization, rather than videos, that can provide a comprehensive view of the bladder and allow expert or outside review and referencing. At follow-up, accurate cystoscopic location information with documentation of pathological findings relative to landmarks in the bladder could directly aid re-examination of specific locations based on previously generated bladder maps. Additionally, while the bladder map orientation in these studies was based on the first frame in the sequence, in practice this could be standardized as beginning with an intravesical landmark.

One challenge of creating a whole bladder map was ensuring that the operator comprehensively swept the cystoscope over the entirety of bladder. The preclinical and clinical images presented in this study have apparent gaps, which may be due to incomplete sweeps or, potentially, shortcomings of the algorithms. Gong et al. developed a “mosaicking” method that allowed for generation of a stitched image using low-quality and few-feature medical images with applications in the retina and elsewhere^21^. Likewise, Phan et al. using a Structure from Motion (SfM) approach and optical flow techniques to accommodate sparse or distorted features^20^. Future iterations of this algorithm could explore such techniques to address this unmet technical need.

Soper et al. introduced a “bladder surveillance system” that coupled an image stitching algorithm, an ultrathin and flexible endoscope, and robotics to improve standardization and ensure full video coverage of the bladder^6^. This type of technology could allow for the collection of cystoscopic images without requiring the physical presence of a urologist. Still, the introduction of such specialized equipment may be cost-prohibitive in resource-scarce settings or low-middle income countries.

There are multiple limitations to our study, including the retrospective application to only a single clinical case. The robustness of the algorithm will need to be further developed, validated, and tested prospectively in clinical cases. For future clinical implementation, recommendations and adjustments may have to be made to standardize the use of the technology. For instance, urologists may have to fill the bladder to a specified volume, or to correlate different maps of the same patient and accommodate patient movement and non-rigid behavior of the bladder, elastic registration may be required. Further clinical investigations could help to determine the feature density required for the algorithm to perform sufficiently to enable tracking and image stitching. Good image quality and feature identification is a requirement to use the technology. Our results suggest that when > 90% of image frame have quality features then the algorithm will successfully stitch a bladder map, yet the limits require further exploration. Additionally, while comparison of a resected bladder to the stitched bladder atlas would be insightful, overcoming the lack of identifiable vasculature post-mortem, which is the primary feature used for image stitching, and the altered shape where the in vivo bladder is distended, is challenging. Direct comparison of rigid and flexible cystoscopes would also help to define limitations of the algorithm and systems approach.

In conclusion, vision-based image stitching, navigation, and referencing can make use of mucosal landmarks to successfully orient and guide the urologist during repeat cystoscopy, both in vitro and in vivo. An image stitching algorithm using a conventional cystoscope can generate a composite map of the whole bladder and be prospectively applied to cystoscopic procedures, potentially facilitating performance metrics in serial procedures in a recurrent or metachronous disease such as bladder cancer. This could prove to be a cost-effective approach to navigation that could be deployed in resource-scarce settings, without the requirement of robotic, electromagnetic, or fiberoptic tracking hardware, and could enable less skilled operators, facilitating a more standardized procedure.

## Materials and methods

An algorithm was developed to generate 2D maps of stitched video frames for localization and tracking of cystoscopic imaging. The algorithm was evaluated on a 2D model, a 3D anthropomorphic bladder phantom, in vivo in swine, and retrospectively with human patient clinical imaging.

### Bladder image stitching algorithm

The algorithm was developed to stitch sequential images from cystoscope videos by matching image surface features (Fig. 1). The sequential images were transformed, compensating for translation, rotation, and zoom of the cystoscope, and a 2D bladder map was constructed. Subsequent cystoscopic videos were used to detect matched image features in the original bladder map, with the goal of defining the location and orientation to a point of interest.

To detect features in each cystoscopic frame and calculate the frame transformation, “Speeded Up Robust Feature” (SURF) and “Random Sample Consensus” (RANSAC) were used. SURF was used to detect features in the current ($\:{Frame}_{Mi}$) and the next sequential cystoscopic video frame ($\:{Frame}_{M(i+1)}$), which could coincide with mucosal features such as vessels. If enough features (≥ 6) were found in each frame, a brute-force feature matcher and RANSAC were used to calculate the affine transformation ($\:{H}_{{M}_{i}}^{{M}_{(i+1)}}$). If an insufficient number of shared features were found between consecutive frames, the transformation of the prior frame was applied (Fig. 2a).

The bladder map orientation was initialized by the first frame ($\:{M}_{0}$). Then, the transformation calculated by feature detection was applied to the next consecutive frame ($\:{M}_{i}$). The bladder map was resized based on the applied transformation, and the process was repeated for all frames in the video (Fig. 2b).

Drift correction was applied to bladder maps to adjust and re-calibrate the transformation (Fig. 2c). In this transformation, similar features were identified between spatially adjacent frames, rather than temporally sequential frames.

### Revisit algorithm

A “revisit” referencing algorithm was developed to enable cystoscopic re-evaluation of the bladder by guiding orientation of the new cystoscopic view, based on the original bladder map (Fig. 2d). Features of the first frame of the “revisit” cystoscopic video ($\:{Frame}_{v0}$) were detected by SURF and a search was conducted for matching features in the original cystoscopic bladder map. A transformation of the “revisit” cystoscopic frame to the bladder map was found, and the orientation of the frame was indicated. The process was repeated for each additional frame in the “revisit” cystoscopic video, to enable referencing.

### Bench development in 2D and 3D models

The accuracy of camera calibration, whole bladder stitched image generation, and the ability to navigate from stitched images was tested using a 2D bladder image and a 3D printed bladder phantom (Fig. 3). A single-use flexible cystoscope (Neoflex, Neoscope Inc., San Jose, CA) with two degrees of freedom was used to image the 2D and 3D models, which both contained unique vascular surface patterns to provide contrast for image feature matching. Each frame had an area of 640 × 426 pixels and 96 dpi. The cystoscope camera was calibrated once before phantom image collection with a 5 × 7 checkerboard (10 mm for each square) to compensate for image distortion caused by the lens curvature (Fig. 3b, top). Single images were acquired with the camera placed at approximately 20 different positions and angles relative to the checkerboard. The intrinsic and extrinsic parameters of the camera were calculated using a standard camera calibration method^22^ (Fig. 3b, bottom).

3D image accuracy was tested using an idealized, rigid bladder phantom (500 mL, 11 × 8 × 7 cm) which was 3D printed (Ultimaker S3, Geldermalsen, the Netherlands). The interior was painted with vascular patterns, and the area was subdivided into 9 regions which were approximately equal in size (Fig. 3e). Two perpendicular sweep patterns were performed by inserting the cystoscope at the approximate urethral orifice and articulating the cystoscope tip to cover half of the bladder phantom as illustrated in Fig. 3g.

The number of images per sweep, the initial stitching time (the processing time to stitch the initial map from the first image to the last), the drift correction time, and the number of images where no transformation was found were recorded. All processing and computations were performed on an MSI PC, with a Windows 10 operating system, 2.2 GHz CPU, and 16.0GB RAM.

### Preclinical evaluation in swine

To evaluate the technology in a controlled and systematic setting, one female Yorkshire domestic swine (Oak Hill Genetics, Ewing, Illinois) weighing 75 kg was studied under a protocol approved by the Institutional Animal Care and Use Committee, was reported following the ARRIVE guidelines and in compliance with the Public Health Service (PHS) Policy on Humane Care and Use of Laboratory Animals (policy). The subject was sedated with intramuscular ketamine (25 mg/kg), midazolam (0.5 mg/kg), and glycopyrrolate (0.01 mg/kg) and anesthetized with propofol (1 mg/kg intravenously). The subject was intubated and maintained under general anesthesia with isoflurane (1–5%) throughout the procedure. A disposable cystoscope (AMBU Inc., Columbia, MD, image size 360 × 360 pixels, 96 dpi) was inserted into the bladder and used to fill it with saline. Intravesical videos were acquired using at least two different systematic patterns of cystoscope motion. At the conclusion of the study, euthanasia was performed under general anesthesia by intravenous administration of Beuthanasia-D (pentobarbital sodium 390 mg/mL and phenytoin sodium 50 mg/mL). A 2D bladder map was created using stitched images and was qualitatively compared to still images of the excised bladder.

### Clinical evaluation

The feasibility of bladder map stitching was retrospectively assessed from a cystoscopy video of a human patient undergoing evaluation for microscopic hematuria or bladder cancer. Institutional Review Board approval was obtained with waiver of written informed consent as specified in 45 CFR parts 46.116 and 46.117 at the National Institutes of Health and all studies were performed in accordance with the Declaration of Helsinki. A rigid cystoscope (Karl Storz, Tuttlingen, Germany) was utilized. After the completion of the clinical cystoscopic evaluation, the bladder was fully distended and revisited with the cystoscope. This evaluation was acquired by steadily sweeping the cystoscope in a cranio-caudal pattern, ensuring complete surveillance of the visualizable bladder mucosa. This final bladder evaluation was utilized to create a bladder map. The 96 dpi original images were cropped from 1920 × 1080 pixels to 740 × 740 pixels to enable faster image processing.

## Electronic supplementary material

Below is the link to the electronic supplementary material.

Supplementary Material 1

## Data Availability

The data analyzed during the current study are available from the corresponding author on reasonable request.

## Abbreviations

2D: Two-dimensional
SURF: Speeded-up robust feature
RANSAC: Random sample consensus
